# Genetic Variants of Retinoic Acid Receptor-Related Orphan Receptor Alpha Determine Susceptibility to Type 2 Diabetes Mellitus in Han Chinese

**DOI:** 10.3390/genes7080054

**Published:** 2016-08-20

**Authors:** Yuwei Zhang, Yulan Liu, Yin Liu, Yanjie Zhang, Zhiguang Su

**Affiliations:** 1 Division of Endocrinology and Metabolism, West China Hospital, Sichuan University, Chengdu 610041, China; doczhangyuwei@sina.com; 2 Molecular Medicine Research Center, West China Hospital, and State Key Laboratory of Biotherapy, Sichuan University, Chengdu 610041, China; liuyulan163163@163.com (Y.L.L.); liuyin14@163.com (Y.L.); zhangyanjiehaisong@126.com (Y.Z.)

**Keywords:** *RORA*, type 2 diabetes, gene variation, association, haplotype

## Abstract

Retinoic acid receptor-related orphan receptor alpha (RORA) plays a key role in the regulation of lipid and glucose metabolism and insulin expression that are implicated in the development of type 2 diabetes mellitus (T2DM). However, the effects of genetic variants in the *RORA* gene on the susceptibility to T2DM remain unknown. Nine tagging single-nucleotide polymorphisms (SNPs) were screened by using the SNaPshot method in 427 patients with T2DM and 408 normal controls. Association between genotypes and haplotypes derived from these SNPs with T2DM was analyzed using different genetic models. Allele and genotype frequencies at rs10851685 were significantly different between T2DM patients and control subjects (allele: *p* = 0.009, Odds ratios (OR) = 1.36 [95% Confidence intervals (CI) = 1.08–1.72]; genotype: *p* = 0.029). The minor allele T, at rs10851685, was potentially associated with an increased risk of T2DM in the dominant model, displaying OR of 1.38 (95% CI: 1.04–1.82, *p* = 0.025) in subjects with genotypes TA+TT vs. AA. In haplotype analysis, we observed that haplotypes GGTGTAACT, GGTGTAACC, and GATATAACT were significantly associated with increased risk of T2DM, while haplotypes GATGAAGTT, AGTGAAGTT, and AATGAAATT were protective against T2DM. These data suggest that the genetic variation in *RORA* might determine a Chinese Han individual’s susceptibility to T2DM.

## 1. Introduction

Type 2 diabetes mellitus (T2DM), a complex metabolic disease, has become a major global health threat in recent years. An estimated 360 million people are currently diabetic worldwide, and this is expected to increase up to 439 million by 2030 [1]. In China, the latest statistical data show that the prevalence of diabetes is 9.7% among people older than 20 years [2]. In addition to dietary habits, sedentary lifestyle, psychosocial stress, smoking, and other environmental parameters which are partially responsible for the development of T2DM, familial studies, including those in twins, as well as migration and admixture studies suggest that genetic factors also contribute to the risk of T2DM [3]. Indeed, numerous studies have associated specific genetic variants with the risk of T2DM. Up to now, more than 88 loci have been identified as conferring susceptibility to T2DM, mostly through genome-wide association studies (GWAS) [4]. However, the effect sizes of these loci are small, and they are still not enough to explain the heritability of T2DM.

Retinoic acid receptor-related orphan receptor alpha (RORA), a member of a distinct subfamily of nuclear hormone receptors, regulates gene expression by binding as a monomer to ROR-responsive elements (ROREs) found in target gene promoters [5]. RORA has been identified as controlling the transcription of genes important in the regulation of lipid and glucose metabolism, such as genes encoding apolipoproteins A1, A5 and C3, glucose 6-phosphatase, and insulin [6,7,8,9,10]. A spontaneous mutation consisting of a deletion within the *RORA* gene, that prevents translation of the RORA ligand-binding domain, has been identified in the staggerer mouse (*RORA*^sg/sg^) [11]. In addition to severe neurological disorders, *RORA*^sg/sg^ mice are also characterized in modulating diet-induced obesity, insulin sensitivity and glucose uptake [12,13]. We recently found that the plasma insulin levels were regulated by locus-containing *RORA* on mouse chromosome 9 [14], and identified RORA as a transcriptional activator of insulin [10]. Moreover, *RORA* was identified as a T2DM susceptibility locus in Mexican Americans and Older Order Amish [15,16]. Based on these previous observations, *RORA* is considered as a convincing candidate gene that may contribute to glucose metabolism.

Owing to the critical roles in a number of biological processes, there is significant interest in the identification of ligands that regulate the transcriptional activity of RORA. Cholesterol, cholesterol sulfate (CS) and various oxygenated sterols have been suggested to be the natural ligands for RORA [17,18]. In addition, since the synthetic liver X receptor (LXR) agonist T0901317 has been identified as the first synthetic RORA inverse agonist, several RORA selective ligands have been synthesized, such as the agonist SR1078 and the inverse agonists SR3335 and SR1001 [17,18]. The identification of endogenous and synthetic RORA ligands suggests modulation of this receptor may be therapeutically feasible, so assessment as to whether it may be a T2DM risk allele is even more important. Therefore, the aim of this current study was to explore the possible correlation between genetic variations in *RORA* gene with the susceptibility to T2DM in a Chinese Han population.

## 2. Materials and Methods

### 2.1. Subjects

In this study, a total of 427 patients with T2DM and 408 age and sex-matched healthy controls were recruited from the West China Hospital of Sichuan University. The subjects in both groups were unrelated ethnic Chinese Han individuals. All subjects underwent anthropometric measurements including height, weight, and blood pressure. Body mass index (BMI) was calculated as weight in kilograms divided by height in meters squared. T2DM diagnosis was carried out by authorized physicians in accordance with the 1999 World Health Organization (WHO) criteria and considering fasting plasma glucose (FPG) level of ≥7.0 mmol/L (126 mg/dL) and/or 2-h post-challenge plasma glucose ≥11.1 mmol/L. Patients were excluded from this study if they had a history of other types of diabetes, malignancy, other metabolic disorders, or impaired liver or renal function. The age-matched healthy individuals were volunteers who came to the West China Hospital for physical examination only. The inclusion criteria for controls were as follows: (1) with normal glucose levels; (2) without any other clinical components; and (3) no family history of diabetes indicated in a standard questionnaire.

All subjects gave their informed consent for inclusion before they participated in the study. The study was conducted in accordance with the Declaration of Helsinki, and the protocol was approved by the Ethics Committee (EA1305012, May 4^th^, 2013) of the West China Hospital, Sichuan University.

### 2.2. Biochemical Measurements

Blood samples from each participant were collected into ethylene diamine tetraacetic acid (EDTA) tubes after an overnight fast. The plasma was separated by centrifugation at room temperature. The levels of triglyceride, total cholesterol, low-density lipoprotein cholesterol (LDL-C), high-density lipoprotein cholesterol (HDL-C), and glucose in plasma were measured by using enzymatic kits from Boehringer Mannheim (Indianapolis, IN, USA).

### 2.3. SNP Selection and Genotyping

From the HapMap website [19], we obtained the genotype data of the *RORA* region in the Chinese population. Tagging SNPs with an r^2^ ≥ 0.8 and minor allele frequency (MAF) ≥ 0.1 were selected using the Tagger software (http://www.broadinstitute.org/mpg/tagger/) (Cambridge, MA, USA). There were nine tagging SNPs including rs17270188, rs1898413, rs11638541, rs8033552, rs10851685, rs8041381, rs340002, rs340023 and rs28724570 (Table S1), which captured 127 SNPs from 1 kb region upstream to 1 kb downstream of the gene (Ensembl accession number ENSG00000069667).

We extracted the genomic DNA from the peripheral blood leukocytes using a DNA extraction kit (BioTeke Corporation, Beijing, China). SNPs were genotyped using the SNaPshot method as described previously [20]. Briefly, DNA fragments containing SNPs were amplified by PCR using primers in Supplemental Table S1. After an additional purification step using shrimp alkaline phosphatase and exonuclease I (Applied Biosystems, Carlsbad, CA, USA), 3 µL of the purified PCR products were mixed with 1 µL of the SNaPshot primers (Table S1), and then a single base-pair extension with the SNaPshot multiplex mix was performed (Applied Biosystems). The SNaPshot reaction products were purified by shrimp alkaline phosphatase, and then mixed with GeneScan-120 LIZ internal size standard and Hi-Di formamide (Applied Biosystems). The mixtures were then analyzed on an ABI 3130 Genetic Analyzer (Applied Biosystems). The genotyping data were analyzed by the software GeneMapper 4.0 (Applied Biosystems).

### 2.4. Statistical Analyses

The demographic and clinical data between the T2DM patients and the control subjects were compared using the Student’s *t*-test. The clinical characteristics between the subjects with different genotypes of rs10851685 were compared using the *χ*^2^ test. Statistical analyses were performed in SPSS version 17.0 (IBM, Chicago, IL, USA). *p* < 0.05 of a two-sided significance level was assigned as significant threshold.

The Hardy-Weinberg equilibrium (HWE) for each SNP among cases and controls was tested using two-sided *χ*^2^ analysis. The distribution of genotypes or alleles between the T2DM patients and the controls was compared under dominant, recessive and additive genetic models. The best genetic model for each SNP was determined using Akaike’s information criterion. Odds ratios (OR) and 95% confidence intervals (CIs) were calculated by unconditional logistic regression analyses [21,22]. Multiple testing corrections were carried out using false discovery rate (FDR) [23], which were done using R language package fdrtool (http://strimmerlab.org/software/fdrtool/index.html) (London, UK). *q* values (corrected *p* values) were adjusted with gender, age and BMI. The significance level was set at *q* < 0.05. The power of a statistical test is calculated to avoid making a false negative decision, which is over 0.95 for each SNP. Haplotype reconstruction was performed using SHEsis (http://analysis.bio-x.cn) (Shanghai, China), and only haplotypes with a frequency >3% in at least one group were tested.

## 3. Results

### 3.1. General Characteristics of the Subjects

The baseline characteristics and biochemical features of the study subjects are presented in Table 1. The patients and control subjects did not significantly differ in age, sex, BMI, and LDL levels (*p* > 0.05). Compared to control subjects, the subjects with T2DM showed statistically lower HDL-C levels (*p* < 0.05) and higher levels of glucose, cholesterol, and triglycerides (*p* < 0.05). Meanwhile, significant differences were noted in the systolic and diastolic blood pressure between patients and controls (*p* < 0.05).

### 3.2. Distribution of the SNPs in RORA between T2DM Patients and Controls

The genotype and allele frequencies of each SNP in both T2DM patients and controls are summarized in Table 2. All genotype distributions of the tested SNPs were in accordance with the HWE in patients and control subjects (all *p* > 0.05), suggesting a lack of evolutionary influences on genetic variation in our subjects. rs10851685 exhibited significant differences in allele or genotype frequencies between T2DM patients and control subjects, while other SNPs did not. Compared to the allele A at rs10851685, the allele T is associated with a significant increased risk of T2DM (OR = 1.36, 95% CI: 1.08–1.72, *p* = 0.009). The genotypes distributed significantly different between T2DM patients and control subjects (*p* = 0.029).

### 3.3. Association of Genotypes with T2DM under Different Genetic Models

The maximum power in genetic association studies is reached when the “true” model of inheritance of disease susceptibility loci and the genetic model used in the analysis are concordant. Diverse genetic models including dominant, recessive and additive models were used to compare the genotype frequencies of each SNP between groups, and the best one was determined using Akaike’s information criterion. For each SNP, the minor allele, whose frequency is relatively lower compared to the wild type one, was assumed as a risk allele (Table 2). As shown in Table 3, the minor allele T at rs10851685 was observed to be associated with an increased T2DM risk under a dominant model TA + TT vs. AA (*p* = 0.025, OR = 1.38, 95% CI: 1.04–1.82).

### 3.4. Effects of rs10851685 on the Different Metabolic Parameters

**Figure 1:**
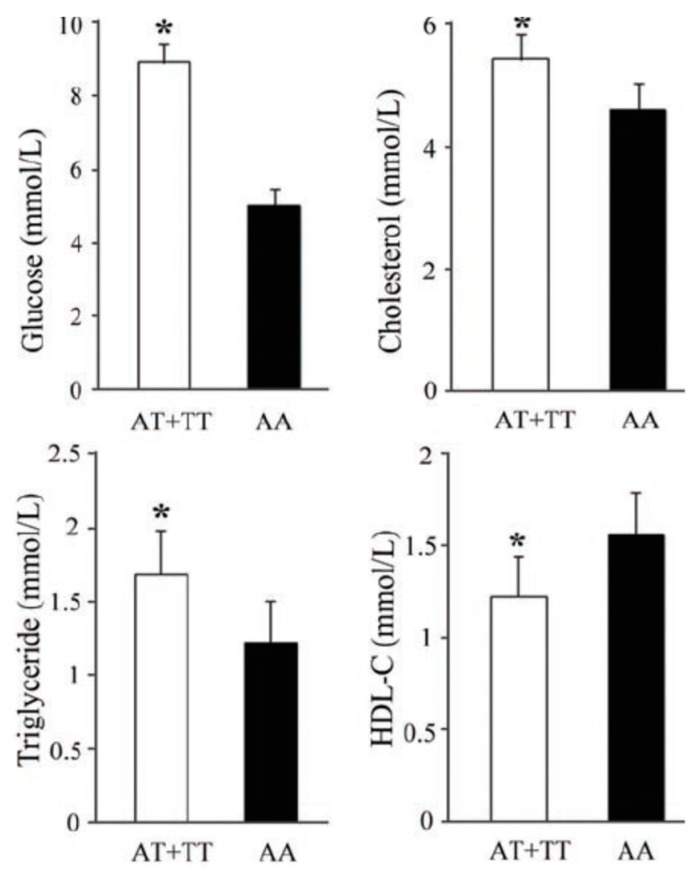
Effects of rs10851685 on metabolic parameters analyzed with covariates gender, age, type 2 diabetes mellitus (T2DM) status and body mass index (BMI). * Statistically significant at *p* < 0.05.

To determine the effects of rs10851685 on clinical characteristics, we further analyzed the associations between the clinical variables and the genotypes of rs10851685 in all participants under a dominant model. As shown in Figure 1, compared to the 510 subjects with AA genotype, the 325 carriers of the risk allele T showed significantly higher concentrations of fasting plasma glucose (8.92 ± 0.54 vs. 5.01 ± 0.47 mmol/L, *p* < 0.05), cholesterol (5.42 ± 0.42 vs. 4.61 ± 0.44 mmol/L, *p* < 0.05) and triglyceride (1.68 ± 0.31 vs. 1.22 ± 0.29 mmol/L, *p* < 0.05), and lower HDL-C concentration (1.23 ± 0.22 vs. 1.56 ± 0.24 mmol/L, *p* < 0.05).

### 3.5. RORA Haplotypes and T2DM

We estimated the frequencies of haplotypes constructed from phased multi-locus genotypes in *RORA*. The haplotypes with a frequency higher than 3% in at least one group were involved in the haplotype analysis (Table 4). Global haplotype association analyses showed that three haplotypes, GGTGTAACT (OR = 2.11, 95% CI = 1.31–3.42, *p* = 0.004), GGTGTAACC (OR = 3.26, 95% CI = 1.78–5.95, *p* = 5.27 × 10^−5^) and GATATAACT (*p* = 1.72 × 10^−6^), were significantly associated with the risk of T2DM. In addition, three protective haplotypes, GATGAAGTT (OR = 0.54, 95% CI = 0.39–0.81, *p* = 0.0001), AGTGAAGTT (OR = 0.490, 95% CI = 0.29–0.91, *p* = 0.018) and AATGAAATT (*p* = 1.16 × 10^−5^), were associated with a decreased risk of T2DM. The overall frequency distribution of haplotype composed of all nine SNPs was significantly different between cases and controls.

## 4. Discussion

In this Chinese Han case-control study, we evaluated the relationships between 9 tagging SNPs in *RORA* region and the susceptibility to T2DM. Our current findings suggest that rs10851685 is associated with the risk of T2DM. In comparison with allele A at rs10851685, the allele T could increase the risk of T2DM under a dominant genetic model. In addition, our haplotype analysis also strongly supports that the genetic variants in *RORA* gene contribute to the susceptibility to T2DM. Some haplotypes with low frequency were found to affect the risk of T2DM dramatically, indicating the complexity of *RORA* gene in the development of T2DM.

Emerging evidence has indicated that RORA plays a critical role in the control of glucose metabolism and the development of T2DM. Steroid receptor coactivator-2 (SRC-2) knockout mice display severe hypoglycemia and abnormal accumulation of glucose in the liver. SRC-2 controls the expression of hepatic glucose-6-phosphatase (*G6Pase*), an enzyme that is crucial for maintaining fasting blood sugar levels by increasing hepatic glucose production and coactivates RORA bound to the RORE on the *G6Pase* gene promoter [24]. Also, it was recently demonstrated that RORA controls the expression and secretion of fibroblast growth factor 21 (FGF21), a hepatic hormone that regulates peripheral glucose tolerance and hepatic lipid metabolism [25]. Over the past few years, it has become increasingly obvious that obesity is a major independent risk factor for developing T2DM [26]. Staggerer (*RORA*^sg/sg^) mice with a natural *RORA* deficiency are protected against age- or diet-induced obesity, hepatosteatosis, and adipose tissue-associated inflammation [12,27]. Several inflammatory and lipogenic genes, including *PLIN2*, *IL1RN*, *OPN*, *CD44*, and *CIDEC*, that are down-regulated in *RORA*^sg/sg^ mice, have been reported to also regulate insulin sensitivity. *RORA*^sg/sg^ mice also exhibited a significantly reduced susceptibility to diet-induced insulin resistance and glucose intolerance compared to wild-type mice [13,27]. The improved insulin sensitivity observed in *RORA*^sg/sg^ mice is also in part attributable to the stimulation in protein kinase B (Akt) signaling [14]. An elevation of Akt expression observed in the skeletal muscle of *RORA*^sg/sg^ mice is correlated with the increased levels of insulin-induced Akt phosphorylation, glucose transporter type 4 (GLUT4) expression, and glucose uptake. In addition, we recently demonstrated that RORA could regulate insulin gene expression directly by binding the gene promoter region [10]. These observations suggest that genetic variants of *RORA* might affect T2DM risk through a combination of multiple pathways.

T2DM is associated with an increase in triglyceride and a decrease in HDL-C concentrations [28]. In our present analysis, we found that the diabetic risk allele T at rs10851685 was associated with significantly increased triglyceride levels and decreased HDL-C levels, suggesting the possible participation of rs10851685 polymorphism in T2DM etiology. Indeed, the *RORA*-deficient staggerer mice exhibited severe dyslipidemia with high triglyceride concentration and low HDL-C level [29]. While HDL-C is mainly responsible for reverse-transporting cholesterol to the liver, it serves as a beneficial effect toward atherosclerosis, which is in contrast with triglyceride. Therefore, RORA may have an influence on the T2DM through participating in lipid metabolism.

The significant variant rs10851685 associated with T2DM in this unrelated case-control cohort occurs within the intron region of the *RORA*, which is usually removed during the gene-splicing process. Although there is no apparent functional change, intronic SNPs may modify gene function by affecting the regulation of gene expression [30]. Various sterols have been identified as the natural ligands for RORA [17,18], so the lipid moieties in Western diets may be endogenous ligands targeting RORA, which may play into the risk allele of rs10851685 and perhaps synergize to enhance T2DM. It is also likely that rs10851685 could just be a surrogate marker for the causal functional variants, which could be present elsewhere in the same gene or in a nearby gene as suggested by analysis of the significant haplotypes, where some SNPs were significantly associated with the T2DM risk as part of a haplotype but not individually (Table 4). Since only SNPs with MAF ≥ 10% were investigated in the present study, it is possible that those rare disease-causing variants were missed. It has been demonstrated that multiple rare variants in genetic architecture are critically important in disease etiology [31].

We are aware that the significant results in the current study could prove to be false positives because of the relatively small sample size and without replication evidence. However, *RORA* has been identified as a T2DM susceptibility locus in Mexican Americans and Older Order Amish [15,16]. *RORA* does not seem to be associated with the susceptibility of T2DM in the reported Chinese cohort studies [31,32,33,34,35,36,37], which could be attributed to the complex genetic structures in the Han Chinese. The Chinese Han population, a seemingly homogeneous population, is actually complicatedly substructured, with the main observed clusters corresponding roughly to northern Han, central Han, and southern Han [38]. The participants in the present study are Southern Han Chinese in Sichuan, while prior Chinese cohort studies are conducted among Chinese Han in Shanghai [35,36,37,39], which are geographically and genetically located central Han [38]. Further studies using larger populations are needed. Even with a larger sample, though, the functional and biological impacts of the described polymorphisms would require further study. Given the multiple functions and the significant roles of RORA in T2DM [40], it is unlikely that *RORA* alone contributes to the sequelae of events in T2DM; rather, genes regulated by RORA, or those that regulate RORA, may influence the disease. Therefore, the investigation of RORA-related pathways and gene networks may lead to a better understanding of the pathophysiology of T2DM.

## 5. Conclusions

To the best of our knowledge, this study is the first to demonstrate that rs10851685 is associated with T2DM in Chinese Hans. Functional analysis determining the allele’s effect on gene expression is necessary, which will identify the molecular basis underlying these genetic predispositions and the potential mechanisms accounting for RORA and T2DM.

## Figures and Tables

**Table 1:** Characteristics of type 2 diabetes mellitus (T2DM) patients and control subjects ^a^.

Variable	Cases (*n* = 427)	Controls (*n* = 408)	*p*
Age (years)	57.37 ± 11.28	58.26 ± 10.51	0.062 ^b^
Sex (Men/Women)	219/208	209/199	0.075 ^b^
BMI (kg/m^2^) ^c^	24.16 ± 2.25	23.28 ± 2.13	0.086 ^b^
SBP (mmHg) ^c^	140.16 ± 18.21	124.41 ± 14.18	**0.007** ^d^
DBP (mmHg) ^c^	83.47 ± 8.68	77.63 ± 9.32	**0.008** ^d^
FPG (mmol/L) ^c^	9.77 ± 1.15	4.86 ± 0.58	**0.001** ^d^
TC (mmol/L) ^c^	5.11 ± 0.92	4.87 ± 0.88	**0.009** ^d^
HDL-C (mmol/L) ^c^	1.22 ± 0.33	1.38 ± 0.37	**0.008** ^d^
LDL-C (mmol/L) ^c^	2.77 ± 0.82	2.69 ± 0.91	0.073 ^b^
TG (mmol/L) ^c^	1.72 ± 0.53	1.21 ± 0.34	**0.003** ^d^

^a^ Data are presented as mean ± standard deviation (SD). ^b^ no significant difference (*p* > 0.05). ^c^ BMI, body mass index; SBP, systolic blood pressure; DBP, diastolic blood pressure; FPG, fasting plasma glucose. TC: total cholesterol; HDL: high-density lipoprotein; LDL: low-density lipoprotein; TG: triglyceride. ^d^ Statistically significant at *p* < 0.05 in bold.

**Table 2:** Distributions of the retinoic acid receptor-related orphan receptor alpha (*RORA*) single-nucleotide polymorphisms (SNPs) in T2DM patients and controls.

SNP	Group		Genotype		HWE ^a^				Allele	
		Number	*p*	FDR *q* ^b^	*p*	Freq. %		*p*	FDR *q* ^b^	OR [95% CI] ^c^
rs17270188		AA/AG/GG				A	G			
	T2DM	94/195/138	0.869	0.871	0.112	44.8	55.2	0.614	0.658	0.95 [0.78–1.15]
	Control	93/190/125			0.204	46.1	53.9			
rs1898413		AA/AG/GG				A	G			
	T2DM	14/112/301	0.772	0.793	0.373	16.4	83.6	0.644	0.681	1.06 [0.82–1.38]
	Control	10/107/291			0.965	15.6	84.4			
rs11638541		TT/TC/CC				T	C			
	T2DM	336/84/7	0.809	0.822	0.512	88.5	11.5	0.541	0.592	1.10 [0.81–1.50]
	Control	327/76/5			0.806	89.5	10.5			
rs8033552		AA/AG/GG				A	G			
	T2DM	17/117/293	0.801	0.814	0.225	17.7	82.3	0.494	0.521	1.09 [0.85–1.41]
	Control	14/106/288			0.280	16.4	83.6			
**rs10851685**		TT/TA/AA				T	A			
	T2DM	26/156/245	**0.029**	**0.038**	0.860	24.4	75.6	**0.009**	**0.015**	**1.36 [1.08–1.72]**
	Control	13/130/265			0.540	19.1	80.9			
rs8041381		AA/AG/GG				A	G			
	T2DM	310/111/6	0.847	0.864	0.262	85.6	14.4	0.745	0.776	1.05 [0.80–1.38]
	Control	299/105/4			0.113	86.2	13.8			
rs340002		AA/AG/GG				A	G			
	T2DM	51/204/172	0.804	0.818	0.421	35.8	64.2	0.586	0.601	1.06 [0.871–1.29]
	Control	43/196/169			0.210	34.6	65.4			
rs340023		CC/CT/TT				C	T			
	T2DM	68/189/170	0.661	0.692	0.206	38.1	61.9	0.421	0.479	1.09 [0.89–1.32]
	Control	56/183/169			0.566	36.2	63.8			
rs28724570		CC/CT/TT				C	T			
	T2DM	107/215/105	0.311	0.358	0.884	50.2	49.8	0.134	0.142	1.16 [0.96–1.40]
	Control	90/200/118			0.762	46.6	53.4			

**^a^** HWE: Hardy-Weinberg equilibrium. ^b^ FDR *q*: false discovery rate *q* value. ^c^ OR: odds ratio; CI: confidence interval. *p* or *q* value of 0.05 is in bold.

**Table 3:** Association between *RORA* SNPs and the risk of T2DM under different genetic models.

SNP	Genetic Model ^a^		*p* Value	FDR *q*	OR [95% CI]
rs17270181	Dominant	(AG + AA) vs. GG	0.183	0.231	1.32 [0.88–2.01]
rs1898413	Additive	AG vs. GG	0.918	0.921	1.01 [0.81–1.26]
		AA vs. GG	0.384	0.413	1.30 [0.72–2.34]
rs11638541	Dominant	(AG + GG) vs. AA	0.222	0.298	1.15 [0.92–1.44]
rs8033552	Recessive	AA vs. (AG + GG)	0.178	0.202	1.34 [0.87–2.05]
**rs10851685**	Dominant	(TA + TT) vs. AA	**0.025**	**0.032**	**1.38 [1.04–1.82]**
rs8041381	Dominant	(AG + GG) vs. AA	0.333	0.402	0.88 [0.58–1.33]
rs340002	Dominant	(AG + AA) vs. GG	0.699	0.748	1.04 [0.86–1.26]
rs340023	Recessive	CC vs. (CT + TT)	0.195	0.243	1.21 [0.91–1.62]
rs28724570	Recessive	CC vs. (CT + TT)	0.057	0.103	1.79 [0.98–3.23]

**^a^** For each SNP, only the best genetic model determined by Akaike’s information criterion (AIC) is provided. The ORs and CIs that are statistically significant are bolded, along with the rs number of the corresponding SNP.

**Table 4:** Frequencies of pairwise haplotype constructed by SNPs in *RORA*.

Haplotype ^a^	Freq. (case)	Freq.	*χ*^2^	Fisher’s *p*	OR [95% CI] ^b^
		(control)			
GGTGTAGTT	0.044	0.031	1.03	0.417	
GGTGTAGTT	0.072	0.056	2.37	0.097	
GGTGTAACT	0.102	0.058	8.38	**0.004**	**2.11 [1.31–3.42]**
GGTGTAACC	0.093	0.024	17.21	**5.27 × 10^−^**^5^	**3.26[1.78–5.95]**
GATATAGCT	0.061	0.06	3.01	0.086	
GATGAGGTT	0.038	0.029	0.08	0.862	
GATGAAGTT	0.179	0.234	13.46	**0.0001**	**0.54 [0.39–0.81]**
AGTGAAGTT	0.033	0.054	5.36	**0.018**	**0.49 [0.29–0.91]**
GATATAACT	0.046	0	19.51	**1.72 × 10^−^**^6^	
AATGAAATT	0	0.034	18.79	**1.16 × 10^−^**^5^	

^a^ The order of SNPs from left to right is rs17270188, rs1898413, rs11638541, rs8033552, rs10851685, rs8041381, rs340002, rs340023 and rs28724570. Only haplotypes with a frequency > 3% in at least one group were listed. ^b^ Only haplotypes distributed significantly differently (*p* < 0.05) were calculated. The OR could not be calculated for the haplotypes GATATAACT and AATGAAATT, because of the zero value in the population.

## Supplementary Materials

The following is available online at www.mdpi.com/2073-4425/7/8/54/s1, Table S1: Nine single-nucleotide polymorphisms (SNPs) in the *RORA* gene in the study.
