# Supplementary material for: Genetic Variants of Retinoic Acid Receptor-Related Orphan Receptor Alpha Determine Susceptibility to Type 2 Diabetes Mellitus in Han Chinese
Source: Genes (Basel). 2016 Aug 20;7(8):54. doi: 10.3390/genes7080054 (PMC4999842; doi:10.3390/genes7080054)
Supplement: Supplementary file 1 [file genes-07-00054-s001.pdf]

Supplementary Materials: Genetic Variants of Retinoic Acid Receptor-Related Orphan Receptor Alpha Determine the Susceptibility to Type 2 Diabetes Mellitus in Han Chinese

Yuwei Zhang, Yulan Liu, Yin Liu, Yanjie Zhang and Zhiguang Su

Table S1. Nine single-nucleotide polymorphisms (SNPs) in the *RORA* gene in the study.

SNPs	Position (GRCh38.p2)	Alleles	Primers (5′–3′)	
			PCR	SNaPshot
rs17270188	60,507,710	G/A	GAAATGGTACCTATTTCACAG<br>GACTTGAATTGTCCTTCCAG	AATATTAGTTATTATTATACTTGAAGCATAA
rs1898413	60,528,610	G/A	GGATCCCTTACAGTGCTTAC<br>CATTACTAGGCAGACTTCTTG	CTACATAACACATTGCAAATGGTCACA
rs11638541	60,539,998	T/C	AGTTCTGGCAGCTAGAACAC<br>GCATTACTGACACTATCTGC	ATGTCTTGCCATTAGAGATGAGACAAGGCAACACCATGCT
rs8033552	60,551,386	G/A	TAACGTCCCTTCCCTGTTGT<br>AGATGGCTCTTTGTACTCCC	TGATTCTGATGCTTTGGCTCATCCTGGTCCCTGCTC
rs10851685	60,563,001	A/T	TTTACTGCCAGTGAGAACCA<br>TGTCCTGATGAGACCAAAAG	GAATGATCCACACTCTCATCTAGTTGCTTTTA
rs8041381	60,570,763	A/G	TAGCAACGTCACCACATCGT<br>TACCACCACTTCACAGAGGT	GATACTTGGCCTTCATGATACGGAG
rs340002	60,580,912	G/A	GGTGGAGTAAGCTGTGATC<br>TACAGAAAATGTGGGTGC	GAGCTGTCAGTCATTTCTTACATCTGAACAACCATGTTTCTTTACAGC
rs340023	60,615,883	T/C	CATGGCAACCAGTTCTTCAG<br>GAGGAACTAAATTCAGCACC	GTTGAGAGGGCTGCTTCGAAAATAACTCTG
rs28724570	60,625,076	T/C	CAGATAGACTTAGGTGTAGG<br>TAAGCCACAGAAGGTCAG	ATATTCCAAATAATGATAGGGCATACTTTTATTCATTGTTCATC
